# The incidence of juvenile rheumatoid arthritis in Quebec: a population data-based study

**DOI:** 10.1186/1546-0096-7-20

**Published:** 2009-11-19

**Authors:** Debbie Ehrmann Feldman, Sasha Bernatsky, Michelle Houde

**Affiliations:** 1 Université de Montréal, Interdisciplinary Research Centre in Rehabilitation, and Public Health Department of Montreal, Pavillon 7077 du Parc, CP 6128, Succ Centre-ville, Montréal, Qc H3C 3J7, Canada; 2 McGill University, Montreal, Canada Division of Clinical Epidemiology, Research Institute of the McGill University Health Centre, Royal Victoria Hospital, V Building, 687 Pine Avenue West, Montreal, QC H3A 1A1, Canada; 3 Public Health Department of Montreal, Agence de la santé et des services sociaux de Montréal, Direction de Santé Publique, 1301 rue Sherbrooke Est, Montréal (Qc) H2L 1M3, Canada

## Abstract

**Objective:**

To determine the population incidence of juvenile rheumatoid arthritis (JRA) in Quebec.

**Methods:**

We obtained data from Quebec's physician claims database. Incident cases were defined as having a visit for JRA in 2000, no visit in the previous 3 years, a confirmed diagnosis by an arthritis specialist, or having ≥ 2 visits to any physician for JRA, ≥ 2 months apart but within 2 years.

**Results:**

Cumulative incidence of JRA was 17.8/100,000. Mean age at diagnosis was 9.8 ± 4.6 years, 68% were female and more persons were diagnosed in winter. Subjects had a median of 10 medical visits over the first year.

**Conclusion:**

Our population based incidence estimate was similar to others. Children and adolescents with JRA are heavy users of medical care. Additional study of environmental or climate- related triggers may be warranted.

## Background

Juvenile idiopathic arthritis (JIA) is a group of inflammatory arthropathies that includes three main groups: juvenile rheumatoid arthritis (JRA), juvenile spondyloarthropathies (or enthesitis-related arthritides) and juvenile psoriatic arthritis [1]. The etiology is unknown and the pathophysiology is poorly understood. Due to the effects of chronic inflammation on joints, affected children often suffer permanent disability, impaired functional status, and poor quality of life in their adult lives [2]. However, recent advances in treatment appear promising and early drug therapy combined with rehabilitation can optimize outcomes [3].

Epidemiologic studies have noted a relatively wide incidence of juvenile arthritis ranging between approximately 10 and 23 per 100,000 persons, with a known female predominance [4-7]. Variations in estimates may be due to differences in diagnostic criteria, case ascertainment, or to regional and time differences [8,9]. Most of the incidence studies to date have relied only on physician reporting. We sought to determine the incidence of JRA in the province of Quebec, Canada using a physician reimbursement database that covers the entire population. We report incidence for JRA (as opposed to JIA) since the database contains a well-defined code for this diagnosis.

## Methods

We obtained data from a physician claims administrative database covering all residents of the province of Québec (the Régie de l'assurance maladie du Québec: RAMQ), after receiving permission from the Commission d'accès à l'information du Québec (Quebec Access to Information Commission). Our study population consisted of all children, 16 years and under, who had a visit to a physician for JRA (International Classification of Diseases-9 code 714) in the year 2000. Data were available for these children and adolescents for the period between January 1997 and June 2003.

Among the 842 children and adolescents with at least one physician visit coded for JRA in the year 2000 (which we considered as suspected cases of JRA), we established that 523 had had no prior physician visits for JRA in the preceding 3 years. For a more specific case-definition of incident JRA among these 523 subjects with suspected JRA, we included only incident cases that had either been diagnosed by a pediatric arthritis specialist, or who had ≥ 2 visits for JRA at least two months apart but within a two year span [10]. We included as pediatric arthritis specialists all rheumatologists, internists or pediatric immunologists who had provided care for JRA patients in a pediatric hospital, and all pediatricians who over the study period had recorded over 50 JRA patient visits.

We report the cumulative incidence of juvenile arthritis for the year 2000. In addition, we describe incidence by sex, season, and socio-economic status. Socioeconomic status was based on a validated indicator that utilizes postal code to estimate neighborhood socioeconomic status and provides an ecological index of material and social deprivation [11]. We dichotomized socioeconomic status at the top two quintiles versus the lower three. We also describe medical service use for the incident JRA cases.

## Results

**Figure 1:**
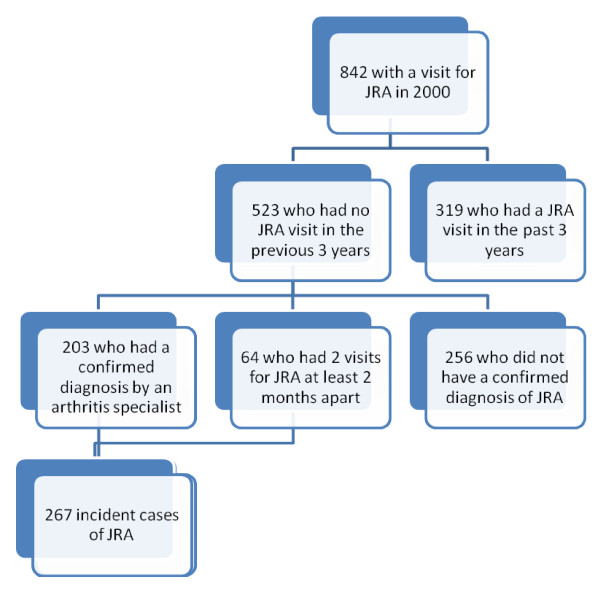
**Definition of incident JRA cases in Quebec**.

We identified 267 new cases of JRA in 2000, in the province of Quebec - 203 were diagnosed by an arthritis specialist and 64 by a pediatrician, family physician or another specialist (Figure 1). Based on census data, there were approximately 1.5 million Quebec residents aged ≤ 16 in 2001, so we estimate the incidence of JRA in 2000 as 17.8 cases per 100,000 (95% confidence interval: 16.05/100,000, 19.55/100,000). The mean age at diagnosis was 9.8 years (standard deviation 4.56, median 10.0).

Descriptive statistics are recorded in Table 1. There was a trend (P < 0.01) suggesting more incident cases in the winter season (December 21-March 20)[12].

**Table 1:** Description of Incident Cases of JRA

N = 267	n	%
Girls	181	67.8

High socioeconomic status	115	43.9

Season of diagnosis		
Winter (December 21-March 20)	79	29.6
Spring (March 21-June 20)	60	22.5
Summer (June21-September 20)	63	23.6
Fall (September21-December 20)	65	24.3

In the first year post diagnosis, the median number of total doctor visits was 10 (inter-quartile range (IQR) 6-17). Medical visits are described in Table 2.

**Table 2:** Medical visits for incident cases of JRA (n = 267), over the first 12 months following initial diagnosis

	12 months post diagnosis	
	**Mean (SD)**	**Median (IQR)**

All medical visits	17.4 (28.4)	10.0 (6.0-17.0)

Arthritis specialist	4.3 (5.6)	3.0 (1.0-5.0)

Pediatrician or Family Physician	6.3 (9.6)	3.0 (2.0-7.0)

Ophthalmologist	1.8 (1.7)	1.0 (0.0-2.0)

Radiologist	0.8 (1.9)	0.0 (0.0-1.0)

Other specialist	4.9 (15.4)	1.0 (0.0-4.0)

## Discussion

Our study produced the first population-based cumulative incidence for JRA in Quebec. This was estimated at 17.8 cases per 100,000 Quebec residents aged ≤ 16, a figure within the range of other incidence studies of juvenile arthritis to date. We noted a clear female preponderance of JIA which is in keeping with known demographics,[5,13]. and mean age at diagnosis was also consistent [5,7].

Our results are similar to the JRA incidence rate of 19.5/100,000 in Finland [5], JIA incidence rate of 21.7/100,000 in Estonia,[7] and 15/100,000 in the Nordic countries [6]. Although we have not validated our algorithm with patient clinical data, the coherence of these rates with other studies that use clinical data suggests that administrative data may be useful to study the epidemiology of JRA. Further, our study covered the entire population of the province of Québec including those living in rural and outlying regions.

According to our data, there was a trend towards first diagnosis during the winter months. One study from Israel has suggested subsets of JRA may be more common in winter and spring, [14] although this was not seen in a large Canadian study [15]. Unfortunately, we could not distinguish between specific JRA sub-types from our administrative data.

Children and adolescents who are *newly *diagnosed with JRA appear to be heavy users of medical care, on average experiencing 17.4 physician visits in the first year post-diagnosis. In comparison, 2004 National Ambulatory Medical Care Survey data suggested an average of 2.4 physician visits per year among children aged ≤ 15 years [16]. Brunner et al., in a sample of prevalent JRA patients, reported similar figures to ours in terms of average rheumatology visits (between 4.4 and 5.8 visits per year) and ophthalmology visits(between1.6 to 2.0) [17]. A British study indicated that children with asthma had, on average, 3.4 to 4.4 annual visits for asthma to out-patient general practice [18]. Although these figures are comparable to ours with respect to visits for JRA to an arthritis specialist, total medical visits and pediatric or family physician visits are higher in our cohort, possibly indicative of higher medical care use by children with JRA.

Since our study relies on data from an administrative database, there are important potential limitations. There is no indication of severity or type of arthritis- factors which influence medical visits and costs [19]. Nevertheless, our study does cover the entire population of the province of Quebec and provides estimates of JRA incidence and medical care use on a population level. Another limitation is that the incidence may have been over-estimated if children and adolescents with previously diagnosed disease moved to Quebec and were included as incident cases in our study.

In conclusion, our population-based study of children and adolescents in Quebec estimated 17.8 incident JRA cases per 100,000 which is similar to physician-reported studies. We confirmed that these individuals are heavy users of medical care, which emphasizes the impact of arthritis in the young. As well, the suggestion of seasonal trends may mean that additional study of environmental triggers in JRA is warranted.

## List of abbreviations

JIA: Juvenile idiopathic arthritis; JRA: Juvenile rheumatoid arthritis; SD: Standard deviation; IQR: Inter-quartile range.

## Competing interests

The authors declare that they have no competing interests.

## Authors' contributions

DEF was responsible for carrying out the study, involved in conception and design, acquisition of data, analysis, interpretation and writing of the manuscript.

SB helped with conception and design, acquisition of data, analysis, interpretation and critical revision of the manuscript.

MH was involved in data analysis, interpretation, and critical revision of the manuscript.

All authors approved the final submitted version of the manuscript.
